# Induction of Circularly Polarized Electroluminescence from an Achiral Light-Emitting Polymer via a Chiral Small-Molecule Dopant

**DOI:** 10.1002/adma.201204961

**Published:** 2013-04-02

**Authors:** Ying Yang, Rosenildo Correa da Costa, Detlef-M Smilgies, Alasdair J Campbell, Matthew J Fuchter

**Affiliations:** Department of Physics and the Centre for Plastic Electronics, Imperial College LondonSouth Kensington campus, London, SW7 2AZ, United Kingdom E-mail: alasdair.campbell@imperial.ac.uk; Department of Chemistry, Imperial College London, South Kensington CampusLondon, SW7 2AZ, United Kingdom E-mail: m.fuchter@imperial.ac.uk; Cornell High Energy Synchrotron Source (CHESS), Wilson Laboratory, Cornell UniversityIthaca, NY 14853, USA

Organic light-emitting diodes (OLEDs) are devices that utilize an emissive electroluminescent organic semiconductor (OSC) thin film sandwiched between two electrodes. Employing polymers as the OSC is a highly attractive approach due to their easy solution processibility. This results in low cost, large area device fabrication possibilities using printing techniques. Circularly polarized (CP) light is central to a large range of current and future display and photonic technologies, including highly efficient LCD backlights,^1^ optical quantum information processing and communication,^2^, ^3^ and optical spintronics.^4^ There is therefore high interest in constructing CP-light-emitting devices. Whilst the use of wide-band reflective polarizers as passive components in polymer LED (PLED) devices is one means to engineer a CP light output, this results in a relatively complex and thick device architecture, requiring an additional liquid-crystal cell.^5^ The direct generation of CP light from a conventional PLED would be far more favorable in terms of simplicity, compactness, energy efficiency and product cost, and thus there has been significant interest in their development.

The principle strategy to date to fabricate direct CP-light-emitting PLEDs has been the attachment of pendent chiral side-chains onto achiral conjugated polymer backbones. The degree of circular dichroism or CP-emission obtained from such systems is defined by the dissymmetry factor (*g* factor, where |*g*| ≤ 2), with *g*_abs_ describing circular dichroism, *g*_PL_ describing CP-photoluminescence and *g*_EL_ describing CP-electroluminescence. Whilst early successful attempts at using this approach, for example with a chiral-substituted poly(*p*-phenylene vinylene) (PPV), only obtained *g*_EL_ in the region of 10^−3^,^6^ considerable synthesis and device optimization effort has resulted in polymers with *g*_EL_ up to 0.35 (see Supporting Information, Table S1 for comparative data). Subsequent analysis has established the role of the chiral pendent side chain, in aligning the polymer into a chiral arrangement.^7^, ^8^ An elegantly simple and highly translatable alternative approach should be the use of a chiral small-molecule dopant to induce this effect by blending it with a conventional achiral light-emitting polymer (LEP). This would avoid the need for bespoke polymer synthesis and could potentially allow the use of a wide range of device-optimized copolymers emitting across the full visual spectrum. However, whilst a small number of reports have emerged on using relatively large chiral molecular architectures such as polysaccharides,^9^ biaryl compounds,^10^ or chiral solvent mixtures^11^ in order to induce CP-photoluminesence (CP-PL) from conjugated polymers, these approaches or dopants have significant limitations for translation into the solid state. Therefore the fabrication of direct CP-EL emitting devices has not been previously demonstrated by this method. Furthermore, the level of CP-PL observed from these studies was only very low (*g*_PL_ = 10^−2^ to 10^−3^, see Supporting Information, Table S1), thus being far from competitive with the polymers bearing chiral side-chains.

Helicenes are intrinsically helical (and therefore chiral) conjugated molecules based on a spiral of fused carbocyclic or heterocyclic rings^12^ (for example **Figure** **1**a). They can be separated into their right-handed and left-handed enantiomeric forms, which are well known to exhibit strong chiroptical properties, such as high optical rotatory power and strong circular dichroism.^12^, ^13^ This paper concerns our preliminary studies to investigate whether helicenes would act as a chiral dopant and confer their helical shape properties to a conventional achiral LEP, while using their organic semiconducting properties to ensure effective device operation of a direct CP-emitting PLED (structure shown in Figure 1c). 1-Aza[6]helicene (Figure 1a) was initially chosen for this study, in light of the fact that it can be scalably synthetically assembled and separated into its enantiomeric forms.^14^ While certain helicenes have been shown to directly emit low levels of CP photoluminescence in solution^15^ and racemic helicenes used as the sole organic semi-conductor component of non-CP emitting OLEDs,^16^ the approach of using dopant quantities of these compounds to control the CP-emission from a conventional LEPs has not been previously reported. Furthermore, to the best of our knowledge, this particular helicene has not been previously studied with regard to organic semiconductor devices.

**Figure 1:**
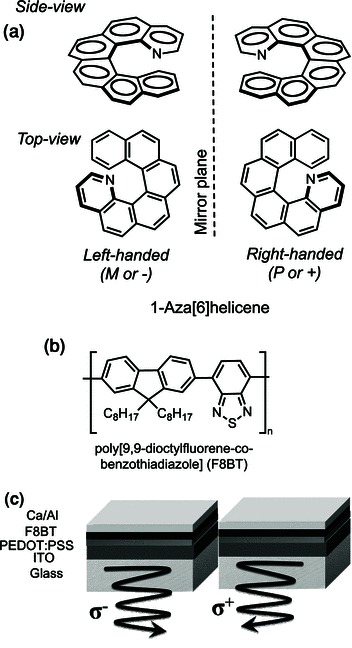
a) Molecular structures of 1-aza[6]helicene right- (*P* or (+)) and left- (*M* or (−)) handed enantiomers. b) Molecular structure of the light-emitting polymer F8BT. c) Single-layer PLED device structure consisting of a 1-aza[6]helicene-doped F8BT thin film sandwiched between a PEDOT:PSS-coated ITO anode and a Ca/Al cathode; right- and left-handed σ^+^ and σ^−^ circularly polarized electroluminescent polymer emission was respectively generated from (+)- and (−)-1-aza[6]helicene-doped devices.

Each 1-aza[6]helicene enantiomer was blended into the conventional PLED material poly[9,9-dioctylfluorene-*co*-benzothiadiazole] (F8BT) (Figure 1b).^17^ A variety of blending ratios (up to 55%) were employed to explore the impact of the helicene additive on the morphology and spectroscopic characteristics of the F8BT thin film. Whilst thin films of pure F8BT were relatively smooth, uniform and featureless, blend ratios of 7% and above resulted in a granular morphology with crystallite sizes ranging from 50 nm to 200 nm (see Supporting Information, Figure S2). Preliminary grazing-incidence wide-angle X-ray scattering (GIWAXS) showed that the blend films contain a novel and highly orientated crystalline or co-crystalline phase, which is crystallographically distinct from that of the pure F8BT or pure helicene thin films, and has a much larger unit cell (see Supporting Information, Figure S3). This previously unknown structure may resemble that of the intercalated or co-crystalline phase of PBTTT and PCBM recently reported.^18^ Two absorption peaks at 325 nm and 450 nm were observed for the F8BT:helicene blend films, with a gradual increase in the 325 nm peak, attributed to 1-aza[6]helicene^14^ (see Supporting Information, Figure S4), with increasing amounts of dopant (**Figure** **2**a). An emission peak was observed at 580 nm (Figure 2a), regardless of the percentage of helicene present, solely representing emission from F8BT. As expected, analogous spectra were obtained when the enantiomeric (−)-1-aza[6]helicene was employed (data not shown).

**Figure 2:**
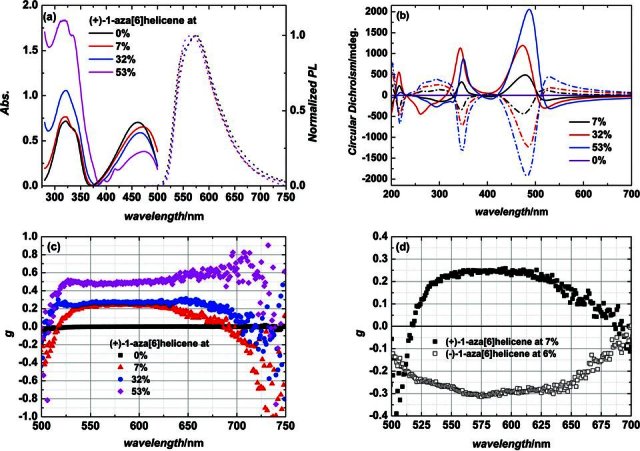
F8BT doped with varying ratios (% by weight) of 1-aza[6]helicene: a) absorption (solid curves) and photoluminescence (PL) (dashed curves) spectra of F8BT doped with (+)-1-aza[6]helicene; b) CD spectra of F8BT doped with (+)-1-aza[6]helicene (solid curves) and (−)-1-aza[6]helicene (dashed curves); c) CP-PL spectra of F8BT doped with (+)-1-aza[6]helicene; d) CP-PL spectra of F8BT doped with 7% (by weight) (+)-1-aza[6]helicene (filled symbols) and 6% (by weight) (−)-1-aza[6]helicene (open symbols).

Circular dichroism measurements of our blend films revealed that thin films of pure achiral F8BT have no CD response (Figure 2b), as expected. Fascinatingly, when just 7% of an enantiopure 1-aza[6]helicene dopant was added, a strong CD signal was observed around 450 nm, which by comparison with the spectra in Figure 2a can be assigned to absorption by F8BT. The strength of the CD response increases with increasing amounts of the helicene additive, with 7% helicene giving a *g*_abs_ value of 0.03 and 53% helicene giving a significantly large *g*_abs_ value of 0.20. This value is similar to those obtained using the chiral side-chain approach (see Supporting Information, Table S1), and is a >25-fold increase over using a polysaccharide dopant.^9^ Similar to the absorption spectra in Figure 2a, another CD peak was observed at 350 nm, which is associated with the known CD response of the helicene additive (see Supporting Information, Figure S4).

Circularly polarized PL (CP-PL) spectra were then obtained for the blend films (Figure 2c). Unsurprisingly, no CP-PL signal was detected from the thin film consisting of pure achiral F8BT. A small amount (7%) of enantiopure 1-aza[6]helicene dopant leads to a big CP-PL response of the F8BT film, with the *g*_PL_ value exceeding 0.2. The CP-PL spectra spans a broad wavelength range from 520 nm to 675 nm and overlaps well with the PL spectra. Increasing the 1-aza[6]helicene blending ratio results in improvements of the *g*_PL_ factor, up to a significantly high value of 0.5 for the 53% helicene blend. Such a value is comparable or better than the *g*_PL_ factors achieved in other studies, which take dramatically different approaches to those disclosed herein (Supporting Information, Table S1). Taken together, the high *g*_PL_ and *g*_abs_ factors, and the doped and undoped PL spectra (Figure 2a), all strongly suggest that the helicene dopant preorganizes the polymer into a chiral structure.

The significant chiroptical response of conductive polymers bearing pendent chiral side chains has been reported to originate from a liquid-crystalline (LC) cholesteric arrangement of the polymer chains in the film,^19^ and this results in a*g* factor which is strongly dependent on film thickness (up to the LC helical pitch length of typically 100 to 200 nm). The film thickness of our blend was therefore varied to determine the effect it would have on the observed chiroptical response. Using an F8BT blend containing 6–7% of 1-aza[6]helicene and altering the spin coating speed, gave films of final thicknesses from 15 nm to 280 nm. The impact of film thickness on the *g*_PL_ factor for thicknesses between 90 nm and 280 nm is negligible (see Supporting Information, Figure S5). At thicknesses below 90 nm, the PL response was too weak to be accurately quantified. Optical microscopy using crossed polarizers was carried out on all film thickness between 15 nm to 280 nm. In all cases, isotropic films were observed (see Supporting Information, Figure S1). AFM was conducted, and a similar granular nanoscale morphology was observed for all films regardless of thickness (see Supporting Information, Figure S2). The fact that the observed isotropic structure, nanoscale morphology, and *g*_PL_ factor are independent of film thickness likely negates a standard cholesteric origin of the chiroptical response. Instead we suggest we have a unique chiroptical co-crystalline phase, as supported by the GIWAXS data (see Supporting Information, Figure S3). It has been previously proposed that a cholesteric origin is required to give *g* factors above 10^−3^ in conjugated LC polymers.^19^ The fact we have *g*_PL_ factors of 0.2–0.5 indicates that appropriate chain packing on much smaller scales (2.36 to 4.7 nm (see Supporting Information, Figure S3) as opposed to 100 to 200 nm) may also lead to strong chiroptical effects in conjugated polymers.^16^ Furthermore, as shown in Figures 2b and Figure 2d, the CD and CP-PL spectra of independent blends prepared from either the left-handed (−)-1-aza[6]helicene or the right-handed (+)-1-aza[6]helicene give comparable but opposite responses. This confirms that the origin of the chiroptical response is the handedness of the helicene dopant employed.

Although this method has high practicality for the conversion of a conventional achiral conjugated polymer into a CP-emitting one, it was unclear whether the observed effect was specific to 1-aza[6]helicene, or indeed more general. A thin-film blend of F8BT with an unrelated enantiopure helicene, [7]helicene^20^ was therefore prepared (**Figure** **3**a). Pleasingly, the CP-PL spectra from the 52% [7]helicene-F8BT blend, shown in Figure 3b, were comparable to those obtained with 1-aza[6]helicene at an analogous blending ratio (53%, see Figure 2c), with high *g*_PL_ factors up to 0.5.

**Figure 3:**
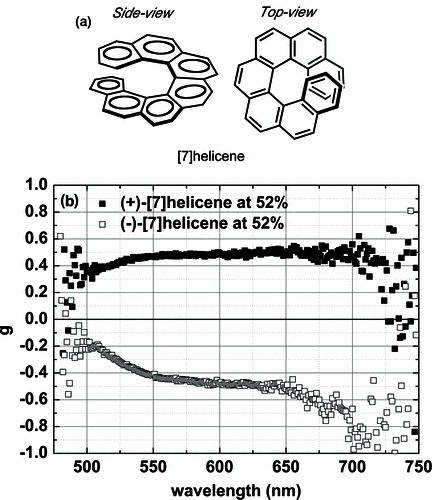
a) Molecular structure of *P* (or +) [7]helicene. b) CP-PL spectra of F8BT films doped with 52% (by weight) (+)- and (−)-[7]helicene.

The most important aspect of our approach was the ability to fabricate the F8BT:helicene blends into functional devices to directly emit CP-EL. Single-layer proof-of-concept PLEDs based on the F8BT blends containing 7% of either the left-handed (−)-1-aza[6]helicene or the right-handed (+)-1-aza[6]helicene were fabricated. Typical current density–voltage–luminance (*J*–*V*–*L*) characteristics obtained for these devices are shown in **Figure** **4**a. To allow comparison, an undoped F8BT device was also fabricated (see Figure 4d). A bright emission achieving 3000 cd/m^2^ was measured for both enantiomeric devices, with an efficiency of 1.1 lm/W. Although there is a slight increase in the turn-on voltage and a decrease in the maximum brightness compared to the reference device, the *J*–*V*–*L* characteristics show that the helicene dopant does not dramatically impact PLED operation or performance. Future synthesis should also allow the helicene to become an integral part of the device (e.g., adjusting the position of the HOMO to assist hole injection and transport). The two vibronic peaks at 550 nm and 575 nm in the EL spectrum (Figure 4b) correspond to the emission observed in the PL spectra (Figure 2a). The EL spectrum is also very similar to the reference device (Figure 4d insert). The CP-EL spectra are shown in Figure 4c. These resemble the profiles of the CP-PL spectra (Figure 2c) and are equal and opposite depending on the enantiomer of the helicene used. A *g*_EL_ factor as high as 0.2 was observed, which also corresponds well to that obtained for CP-PL. Indeed, despite very little optimization, this CP-EL value is comparable or better than that obtained with liquid-crystalline chiral side-chain polymers (Supporting Information, Table S1). While it could be envisaged that increasing the amount of the helicene additive would increase this *g*_EL_ factor further, we generally found this to have a detrimental effect on overall device performance, possibly due to the increase domain height of the resultant thin films (see Supporting Information, Figure S2).

**Figure 4:**
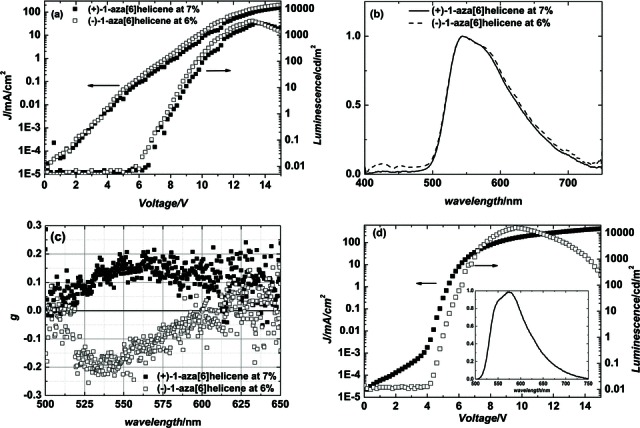
a) Variation of current *J* (circles) and luminosity *L* (squares) with applied voltage *V*. b,c) EL spectra (b) and CP-EL spectra (c) of the PLED device (see Figure 1c) containing a light-emitting layer of F8BT doped with 7% (by weight) of (+)-1-aza[6]helicene (solid symbols) and 6% (by weight) (−)-1-aza[6]helicene (open symbols). d) *J*–*V*–*L* curves of an undoped F8BT PLED with EL spectrum as the inset.

In summary, we have successfully demonstrated a unique and effective approach to directly generate high levels of CP-EL based on doping a conventional achiral polymer with a chiral aromatic molecule. The results disclosed herein represent proof-of-concept, unoptimized devices, and it therefore remains highly probable that further study should result in the development of efficient PLED devices, which emit practically useful levels of CP light directly. The helicenes, being OSCs in their own right, can further be developed as an integral part of the device structure, for example as optimized hole-transport/electron-blocking materials. This strategy should be highly translatable, allowing the production of full-color CP-PLEDs from conventional, well-established PLED materials, using helicene dopants. We believe this will have a significant impact on current display technologies and benefit other disciplines from optical communication to quantum computing.

## Experimental Section

Aza[6]helicene^14^ and [7]-helicene^20^ were prepared as previously reported and separated using preparative chiral HPLC.

*Solution Preparation and Thin-Film Deposition*: Varying proportions of (+)-1-aza[6]helicene and (−)-1-aza[6]helicene (0% to 53% by weight) were blended into a 15 mg/ml F8BT solution (in toluene). Thin films of the blends were deposited by spin coating at 1200 rpm for 60 s onto silica substrates for photophysics or AFM measurements. This gave an average film thickness of 160 nm for all blending ratios (between 6% and 53% 1-aza[6]helicene), measured by Dektak (Veeco). Various film thicknesses from 90 nm to 280 nm were prepared by spin-coating at different spin speeds from 15 mg/ml, 6 wt% doped (+)-1-aza[6]helicene and (−)-1-aza[6]helicene solutions in order to study the effect on CP-PL. Film thicknesses below 90 nm were prepared using a 10 mg/ml solution at different spin speeds.

*Photophysical and Morphology Characterization*: Absorption and PL spectra of the blends at various blending ratios were measured by a Cary 300 UV–Vis spectrometer (Agilent Technologies) and a FluoroMax-3 (Horiba Jobin Yvon), respectively. Thin-film morphology studies were carried out using tapping mode AFM (Veeco multimode).

*PLED Fabrication and Characterization*: Pre-patterned indium tin oxide (ITO) substrates were rinsed in an ultrasonic bath with acetone, isopropyl alcohol (IPA) and deionized water before the deposition of poly(3,4-ethylenedioxythiophene):poly(styrenesulfonate) (PEDOT:PSS) (H.C. Starck GmbH) (50 nm). Thin films of 7% 1-aza[6]helicene: F8BT blend were then spin-coated (1200 rpm for 60 s) onto the PEDOT:PSS-coated ITO substrates. Finally, a 20 nm Ca layer, capped by a 100 nm Al layer was thermally evaporated onto the organic layer to complete the PLED structure. *JVL* characterization was performed using a Keithley 2410 and a Topcon BM-9 luminance meter. PLED emission was assumed to be Lambertian. EL spectra were measured using an Ocean Optics USB 2000 charge-coupled device spectrophotometer.

*Cross-Polarized Microscopy*: An Olympus BX51 microscope was used to performed crossed-polarized microscopy on F8BT: (+)-1-aza[6]helicene blends. The film thickness was varied between 15 nm and 280 nm, fabricated as described above. A 533 nm filter was inserted in between the two linear polarizers to improve the visibility of the images.

*CD, CP-PL and CP-EL Characterization*: The circular dichroism spectra were obtained using a Chirascan-plus CD spectrometer. Left-handed and right-handed CP emission spectra from the blended thin films were collected using a linear polarizer and quarter-wave plate prior to a FluoroMax-3 spectrometer. The background introduced by the polarizer, the quarter-wave plate and the silica substrates were corrected by using the CP-PL results from a blank sample.The dissymmetry factor *g* in the CP-PL spectra was calculated from the equation *g* = 2(*I*_L_ − *I*_R_)/(*I*_L_ + *I*_R_), |*g*| ≤ 2. *I*_R_ and *I*_L_ are the right-handed and left-handed emission intensities respectively. A similar method was used to analyze the CP-EL spectra. Instead of using the FluoroMax-3 as the spectrometer, the left-handed and right-handed EL spectra from the PLED was recorded using an Ocean Optics USB 2000 charge-coupled spectrophotometer.

## Supplementary material

As a service to our authors and readers, this journal provides supporting information supplied by the authors. Such materials are peer reviewed and may be re-organized for online delivery, but are not copy-edited or typeset. Technical support issues arising from supporting information (other than missing files) should be addressed to the authors.
