# Supplementary material for: Induction of Circularly Polarized Electroluminescence from an Achiral Light-Emitting Polymer via a Chiral Small-Molecule Dopant
Source: Adv Mater. 2013 Apr 2;25(18):2624–8. doi: 10.1002/adma.201204961 (PMC3659407; doi:10.1002/adma.201204961)
Supplement: Supplementary file 1 [file adma0025-2624-SD1.pdf]

Copyright WILEY-VCH Verlag GmbH & Co. KGaA, 69469 Weinheim, Germany, 2013.

ADVANCED MATERIALS

Supporting Information

for *Adv. Mater.*, DOI: 10.1002/adma.201204961

Induction of Circularly Polarized Electroluminescence from an Achiral Light-Emitting Polymer via a Chiral Small-Molecule Dopant

Ying Yang, Rosenildo Correa da Costa, Detlef-M. Smilgies, Alasdair J. Campbell, and Matthew J. Fuchter**

SUPPORTING INFORMATION

Induction of Circularly Polarized Electroluminescence from an Achiral Light Emitting Polymer via a Chiral Small Molecule Dopant.

By Ying Yang, Rosenildo Correa da Costa, Detlef-M. Smilgies, Alasdair J. Campbell* and Matthew J. Fuchter*

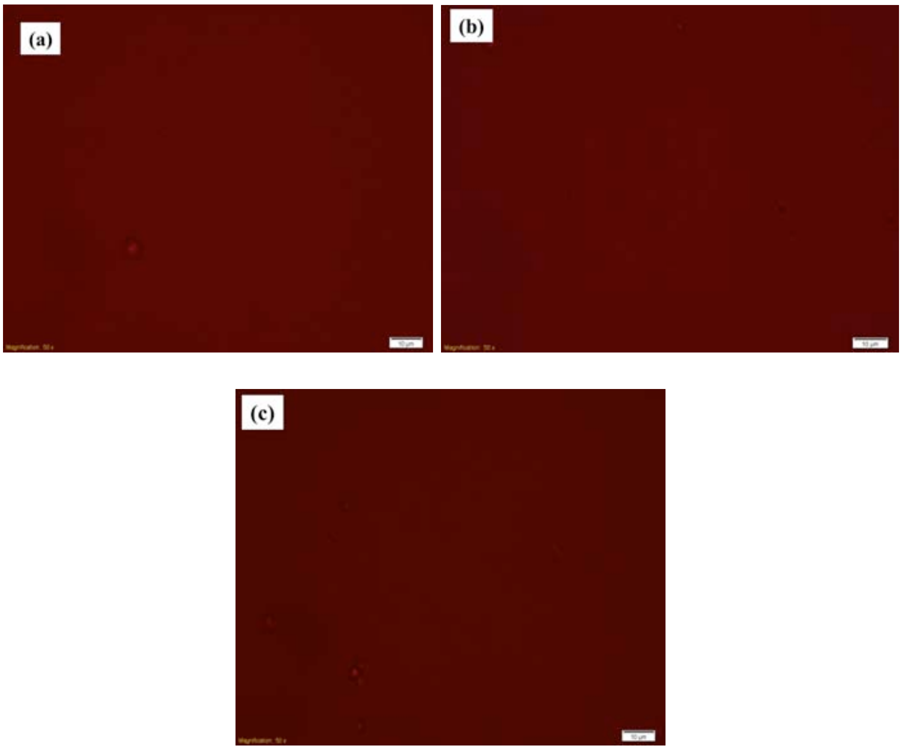

Figure S1. Cross-polarized microscope images of F8BT:(+)-1-aza[6]helicene (7%) blends at film thicknesses of (a) 280 nm; (b) 160 nm; (c) 15 nm. Analogous images were obtained at film thicknesses of 122 nm, 65 nm, 35 nm, and 92 nm (not shown). Images were obtained with the 533 nm filter in between two crossed linear polarizers. A single colour is observed, indicating that the films are isotropic.

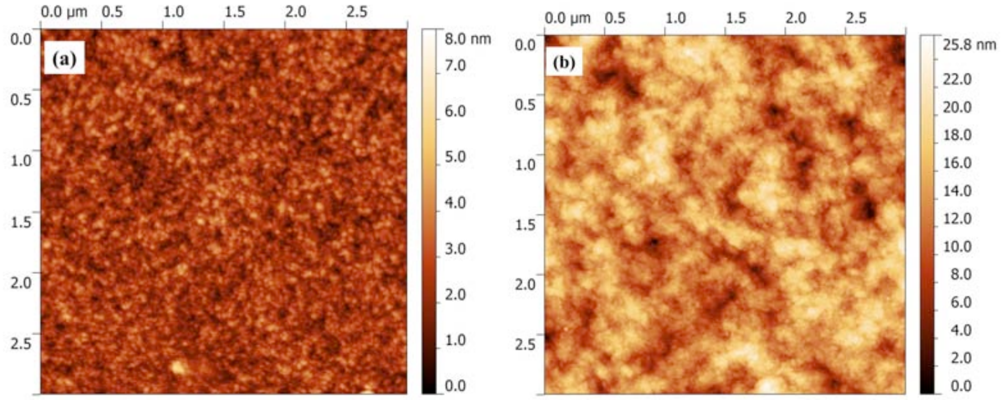

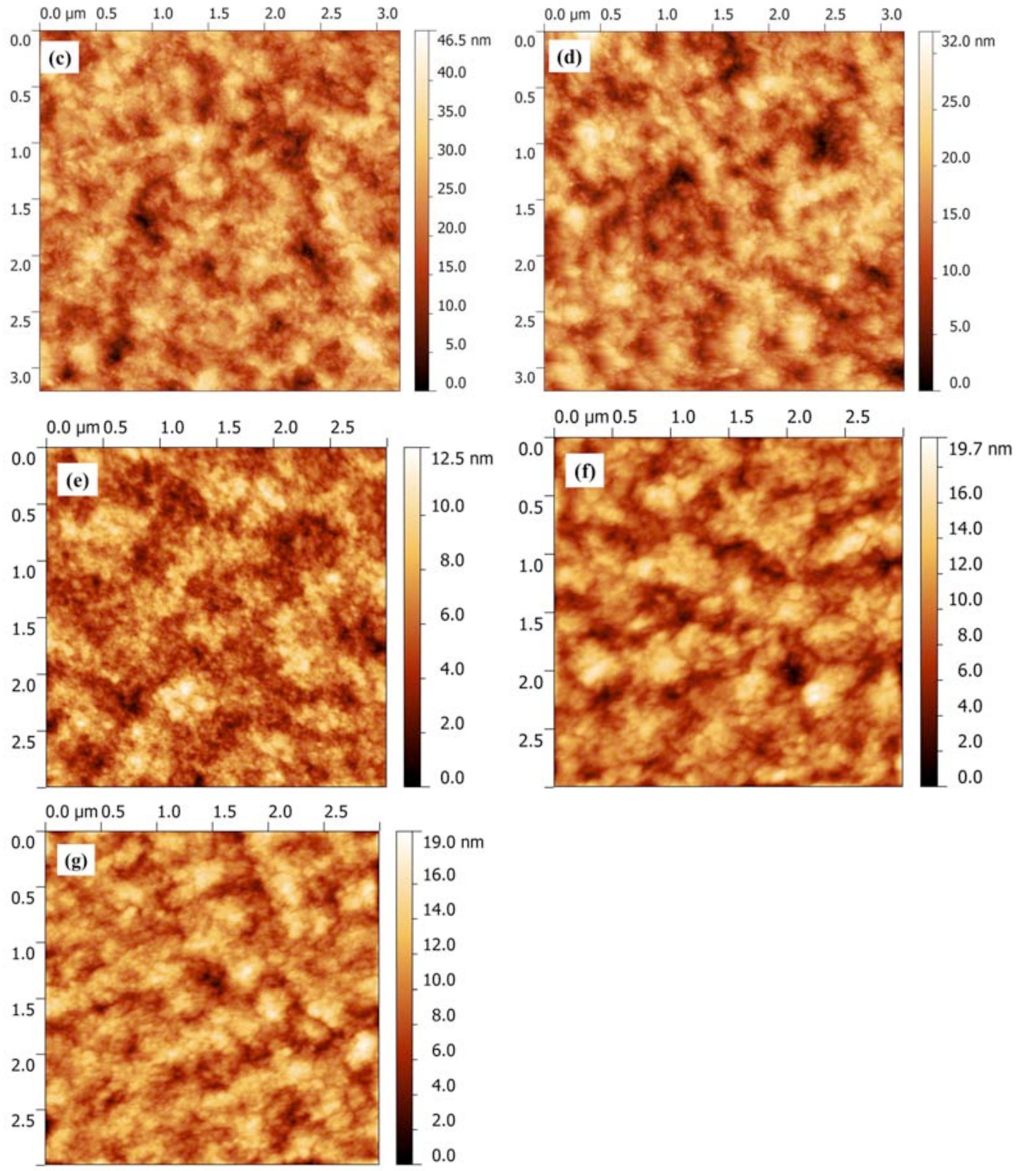

Figure S2. AFM images of 160nm thick thin films of (+)-1-aza[6]helicene: F8BT blends at helicene blending ratio of (a) 0%; (b) 7%; (c) 32%; (d) 55%; AFM images of film thicknesses of (e) 15 nm; (f) 280 nm; (g) 65 nm thick films at a fixed (7%) doping ratio. In the absence of dopant the F8BT film is relatively smooth and uniform, with a surface roughness less than 5 nm. A low doping ratio (7%) has a significant effect on film structure. Small granular crystal domains are observed with sizes ranging from 50 nm to 200 nm. Increasing the doping ratio beyond 7% helicene does not have as much as an effect on the gross morphology of the films, with the crystal domains of the 53% helicene blend only exhibiting a slightly modified size. The most significant change for higher blend ratios was in the domain height, which increased to 50 nm. At a fixed doping ratio (7%), varying the film thickness has little effect on the observed morphology.

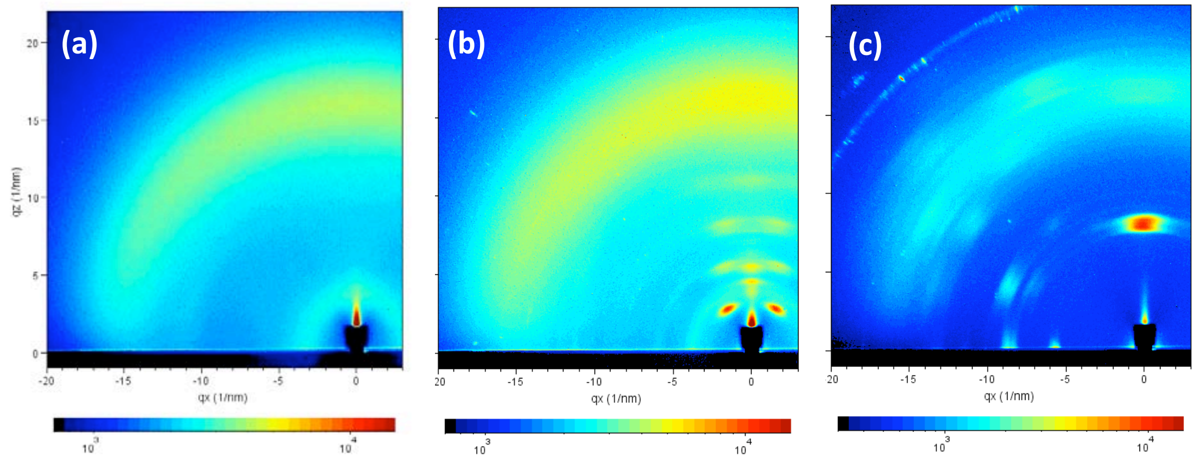

Figure S3. GIWAXS results of (a) pure F8BT; (b) (+)-1-aza[6]helicene: F8BT blend at a ratio of 32% by wt helicene; (c) pure (+)-1aza[6]helicene thin films. The diffraction spots close to the beam stop in (b) correspond to a unique crystal structure with large lattice parameters. These are not correlated with either the F8BT crystal structure reflections (a) or the (+)-1-aza[6]helicene crystal structure reflections (c), as would be the case for a phase-separated blend.

In the pure F8BT pattern (a) the broad arc in the vertical direction with a d-spacing value of 0.42 nm can be associated with the (0 0 4) reflection identified by Donley et al [S10]. The F8BT sidegroup interdigitation c-axis direction therefore lies predominantly normal to the substrate plane [S10, S11]. Initial indexation of the pure (+)-1-aza[6]helicene pattern (c) gives an orthorhombic unit cell with a = 0.73 nm, b = 1.10 nm and c = 1.8 nm. Axis size and orientation and density considerations suggest 4 molecules per unit cell with the helicene stacking direction orientated approximately normal to the substrate plane. Initial analysis of the blend pattern (b) identifies in-plane and normal d-spacings. The in-plane d-spacing is 4.0 nm, and the normal d-spacing 2.36 nm or 4.7 nm. A unit cell for such a structure would have to be much larger than either pure (+)-1-aza[6]helicene (a = 0.73 nm, b = 1.10 nm, c = 1.8 nm) or pure F8BT (a = 1.47 nm, b = 0.53 nm, c = 1.67 nm [S11]) (both of the pure lattices are orthorhombic, Z = 4). The observed d-spacings suggest a unit cell with a volume at least 4 to 8 times greater than the pure phases. Given that both pure phases already have 4 molecules or repeat units per unit cell, this indicates a very complex unit cell containing 10 or more molecules / repeat units.

GIWAXS experimental details. A bending magnet source in combination with multilayer optics supplied an intense x-ray beam with a wavelength of 0.115 nm and 1.5 % bandwidth. The x-ray beam was collimated to a size of 0.5 mm horizontally and 0.1 mm vertically using a slit system. Films prepared as above were placed on a 5-axes goniometer and lined up such that the incident beam impinged on the sample at a typical angle of 0.15 deg. Scattered x-rays were detected with a CCD camera placed 101 mm from the sample. The intense reflected beam was blocked with a 1.5 mm tantalum rod midway between sample and camera. At an effective camera size of 40 mm, the camera could register x-rays with wave vectors up to 20 nm^{-1} corresponding to minimum *d*-spacings of 0.3 nm. The camera was calibrated using a cerium oxide standard.

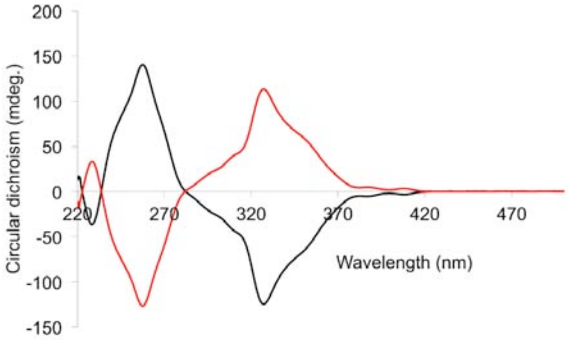

Figure S4. CD spectra of (+)-1-aza[6]helicene (red) and (-)-1-aza[6]helicene (blue) in dichloromethane solution.

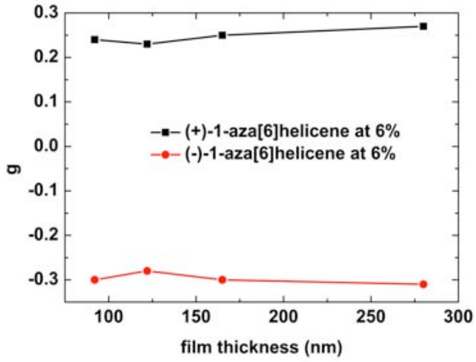

Figure S5. The dependence of *g* factor on film thickness for (+)-1-aza[6]helicene:F8BT and (-)-1-aza[6]helicene:F8BT blends at 6% 1-aza[6]helicene by wt. PL for films below a thickness of 90nm was too weak to be accurately measured.

Table S1. Selected state-of-the-art conjugated polymer thin films.

Material	Special processing conditions?	Thin film g_{abs}[1]	Thin film g_{PL}[2]	g_{EL}[3]	λ_{em} (nm)	Ref.
CHIRAL POLYMERS						
**PPV with chiral side-chains**	N.A.	-5.1x10^{-3}	-7.5x10^{-3}	-1.7x10^{-3}	600	6
**PPP with chiral sidechains**	N.A.	0.06	N.D.	N.D.		S1
**PFO with chiral sidechains**	N.A.	0.03 to 0.05	0.02 to 0.066	0.01 to 0.07	420	S2
**PFO with chiral sidechains**	N.A.	-0.15	N.D.	-0.25.	425	S3
**PFO with chiral sidechains**	N.A.	-0.15	-0.28	-0.25	430	S4
**PFO with chiral sidechains**	On LC alignment layer	Up to 0.8[8]	0.75	N.D.	410	8
**PFO with chiral sidechains**	On LC alignment layer	Up to 0.44[8]	up to 0.7.	0.35	420-550	7
**PFO copolymer with chiral sidechains**	N.A.	Up to -0.17	N.D.	N.D.		S5
**PPE with chiral sidechains**	N.A.	Up to 0.38	0.186.	N.D.	443	S6
**PFO copolymer with chiral sidechains**	N.A.	Up to 0.3	Up to 0.2	N.D.	505	S7
**PFO with chiral sidechains**	N.A.	-1.44 x10^{-4}	-0.16	N.D.	490	S8
**Polythiophene with chiral sidechians**	N.A.	4 x10^{-3}	3 x10^{-3}	N.D.	650	S9
ACHIRAL POLYMERS						
Achiral Polymers with Chiral Solvent						
**PFO with chiral solvents**	N.A.	Up to 1.7x10^{-3} (solution[4])	2.3x10^{-3} (solution[4])	N.D.[5]	434	11
Achiral Polymers with Chiral Dopants						
**Polythioph-ene with polysaccar-ide**	N.A.	7.7x10^{-3} (solution[4])	4.5x10^{-3} (solution[4])	N.D.[6]	561	9
**Charged PPP with charged chiral biaryl**	N.A.	Up to 9 x10^{-2} (solution[4])	-2.4 x 10^{-2} (solution[4])	N.D.[7]	415	11
**PFO copolymer with 7% helicene**	**None**	**0.03[8]**	**0.23**	**0.2**	**550-625**	**THIS WORK**
**PFO copolymer with 53% helicene**	**None**	**0.20[8]**	**0.5**	**N.D.**	**550-625**	**THIS WORK**

N.D. = not determined; [1]g_{abs} = g factor of absorbance; [2]g_{PL} = g factor of photoluminescence; [3]g_{EL} = g factor of electroluminescence; [4]thin film data unavailable; [5]Solution processing using high boiling point chiral terpines as solvents likely to be an issue for device fabrication; [6]Insultating polysaccharide additives are likely to have a detrimental effect on device performance; [7]Solution processing of uniform thin films likely to be an issue; [8]Calculated from g_{abs}=CD°/(32.98A_{linear}).

[S1] R. Fiesel, D. Neher, U. Scherf, *Synth. Met.* **1999**, *102*, 1457.

[S2] M. Oda, S. C. J. Meskers, H. G. Nothofer, U. Scherf, D. Neher, *Synth. Met.* **2000**, *111*, 575.

[S3] M. Oda, H.-G. Nothofer, G. Lieser, U. Scherf, S. C. J. Meskers, D. Neher, *Adv. Mater.* **2000**, *12*, 362.

[S4] M. Oda, H.-G. Nothofer, U. Scherf , V. Šunjić , D. Richter, W. Regenstein, D. Neher *Macromolecules* **2002**, *35*, 6792.

[S5] R. Abbel, A. P. H. J. Schenning, E. W. Meijer, *Macromolecules* **2008**, *41*, 7497.

[S6] S. R. Wilson, M. E. Yurchenko, D. I. Schuster, E. N. Yurchenko, O. Sokolova, S. E. Braslavsky, G. Klihm, *J. Am. Chem. Soc.* **2002**, *124*, 1977.

[S7] T. Hirahara, M. Yoshizawa-Fujita, Y. Takeoka, M. Rikukawa, *Chem. Lett.* **2012**, *41*, 905.

[S8] J. M. Yu, T. Sakamoto, K. Watanabe, S. Furumi, N. Tamaoki, Y. Chen, T. Nakano, *Chem. Commun.* **2011**, *47*, 3799.

[S9] G. Lakhwani, G. Koeckelberghs, S. C. J. Meskers, R. A. J. Janssen, *Chem. Phys. Lett.* **2007**, *437*, 193.

[S10] C. L. Donley, J. Zaumseil, J. W. Andreasen, M. M. Nielsen, H. Sirringhaus, R. H. Friend, and J.-S. Kim, *J. Am. Chem. Soc*. **2005**, *127*, 37.

[S11] Y. Li and J. B. Lagowski, *Computational and Theoretical Chemistry*, **2011**, *997*, 157.
